# Supplementary material for: Splice-Junction-Based Mapping of Alternative Isoforms in the Human Proteome
Source: Cell Rep. Author manuscript; Available in PMC 2020 Jan 15. (PMC6961840; doi:10.1016/j.celrep.2019.11.026)
Supplement: 3 [file NIHMS1546469-supplement-3.zip › DF2/PXD006675/EndothelialCells-17-Q13492-STASQVASTWGGFTPSPVAQPHPSAGLNVDFESVFGNK.pdf]

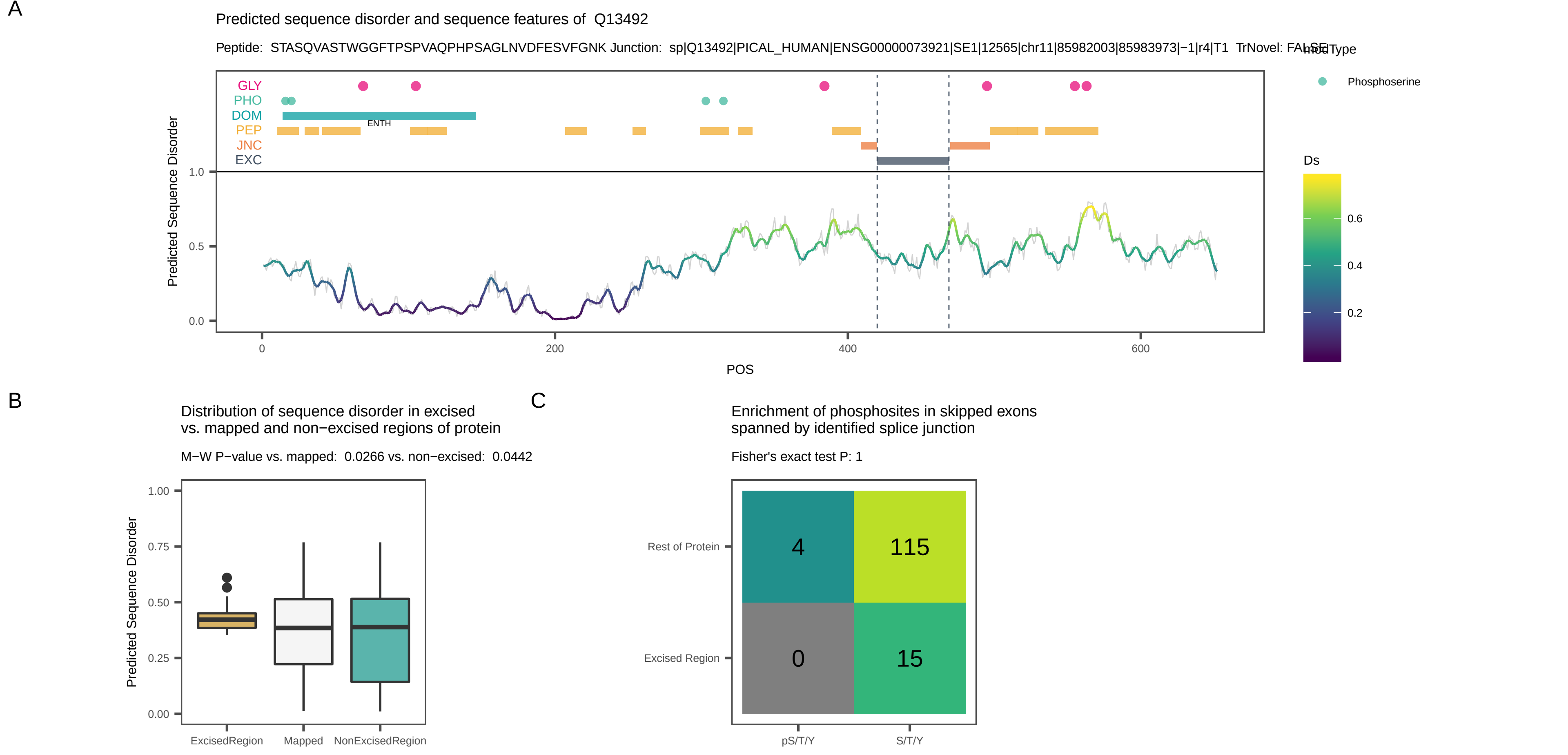

A
Predicted sequence disorder and sequence features of Q13492
Peptide: STASQVASTWGGFTPSPVAQPHPSAGLNVDFESVFGNK Junction: sp|Q13492|PICAL_HUMAN|ENSG00000073921|SE1|12565|chr11|85982003|85983973|−1|r4|T1 TrNovel: FALSE
modType
Phosphoserine
GLY
PHO
DOM
PEP
JNC
EXC
ENTH
Predicted Sequence Disorder
1.0
0.5
0.0
0
200
400
600
POS
Ds
0.6
0.4
0.2
B
Distribution of sequence disorder in excised
vs. mapped and non−excised regions of protein
M−W P−value vs. mapped: 0.0266 vs. non−excised: 0.0442
Predicted Sequence Disorder
1.00
0.75
0.50
0.25
0.00
ExcisedRegion
Mapped
NonExcisedRegion
C
Enrichment of phosphosites in skipped exons
spanned by identified splice junction
Fisher's exact test P: 1
Rest of Protein
Excised Region
4
115
0
15
pS/T/Y
S/T/Y
